# Impact of Treatment Modalities and Fracture Stability on Survival in Thoracolumbar Fractures: A 5-Year Observational Study

**DOI:** 10.3390/jcm14030933

**Published:** 2025-01-31

**Authors:** Reka Viola, Ádám Juhász, Dávid Süvegh, Dániel Sándor Veres, András Gati, Árpád Viola, Mohammad Walid Al-Smadi

**Affiliations:** 1 Department of Psychiatry, Peterfy Sandor Hospital, 1076 Budapest, Hungary; 2 Department of Neurotraumatology, Semmelweis University, 1081 Budapest, Hungarydrgatiandras@gmail.com (A.G.);; 3 Department of Neurosurgery and Neurotraumatology, Dr. Manninger Jenő National Traumatology Institute, 1081 Budapest, Hungary; 4 Department of Biophysics and Radiation Biology, Semmelweis University, 1094 Budapest, Hungary

**Keywords:** thoracolumbar fractures, percutaneous vertebroplasty (PVP), minimally invasive spine surgery (MISS), fracture stability, survival analysis, percutaneous pedicular screw insertion, early recovery after surgery

## Abstract

**Background/Objectives:** Thoracolumbar fractures are a significant health burden, commonly caused by trauma, osteoporosis, or degenerative conditions, and can severely reduce quality of life and survival. These fractures, classified by the AO Spine Classification System, range from stable to unstable and require tailored management strategies. This study aims to evaluate clinical outcomes and survival probabilities in patients aged 50+ with AO A1–A4 fractures, comparing conservative treatment, percutaneous vertebroplasty (PVP), and surgical stabilization, including minimally invasive spine surgery (MISS). **Methods:** This retrospective study analyzed 1356 patients treated for thoracolumbar fractures at Hungary’s largest trauma center (2014–2019). Patients aged 50+ with low-impact trauma-induced AO A1–A4 fractures were included. Fractures were categorized into stable (A1–A2) and unstable (A3–A4) groups. Treatments included conservative management, PVP, and surgical stabilization. Survival probabilities were analyzed using Cox proportional hazards models, and outcomes between open and MISS techniques were compared. **Results:** Spine stability is a crucial factor in determining patient outcomes. MISS enabled stabilization in older patients, reducing hospital stays compared to open surgery (median 6 vs. 10 days). Minimally invasive techniques increased surgical likelihood for unstable fractures, especially in patients over 70 years. Older age and male sex were associated with higher mortality. **Conclusions:** MISS offers reduced recovery time and broader surgical eligibility, making it effective for managing unstable thoracolumbar fractures in older patients. Tailored management strategies are essential for improving survival outcomes, particularly in elderly and frail populations.

## 1. Introduction

Vertebral fractures result from improper axial loading with or without a rotational component and/or distraction/dislocation in the setting of trauma, osteoporosis, infection, metastatic, or other bone diseases [1,2].

Fracture classification systems were designed to guide treatment decisions. Systems evaluate spinal stability, neurological deficit, location, the extent of damage to the bony elements, and the associated ligamentous complexes [3].

Osteoporosis is the most common precipitating factor for vertebral fractures. However, trauma, cancer, chemotherapy, infection, long-term steroid use, hyperthyroidism, and radiation therapy are also known to weaken bones that can lead to compression fractures [4,5].

Vertebral fractures may lead to a reduction in quality of life comparable to diabetes, these injuries have a negative impact on patients’ life expectancy, and the number of fracture-related deaths is comparable to, and in Hungary even exceeds, the number of deaths caused by lung cancer, diabetes, and chronic lower respiratory diseases [6,7,8].

The management of vertebral fractures with the different etiologies involves primarily conservative treatments like pain control, anti-osteoporotic medications, braces, and exercise to stabilize and support healing. Vertebral augmentation, such as vertebroplasty and kyphoplasty, is debated due to mixed outcomes in pain relief and spinal stability, reserved mainly for those unresponsive to conservative treatment. Surgical options are considered for cases with severe instability, pain, or neurologic deficits, employing tailored fusion techniques and fixation methods to manage complications associated with osteoporosis [9].

Extended bed rest for vertebral fracture patients may cause systemic complications, including rapid declines in bone density, muscle strength, thromboembolism, and respiratory capacity, increasing the risk of pneumonia. To counter these effects, early mobilization with adequate pain management and bracing is essential [10,11,12]. Surgical options, primarily posterior pedicle screw fixation, aim to stabilize the spine while minimizing strain on adjacent vertebrae [13,14]. Percutaneous vertebroplasty (PVP), involving cement augmentation, has shown potential for prolonged survival, though treatment protocols vary globally, impacting rehabilitation outcomes and overall patient survival [15].

PVP, may enhance survival in elderly patients with insufficiency fractures, though supporting case–control studies remain limited. Surgical protocols for spinal fractures vary internationally; for example, Germany surgically stabilizes over 96% of AO A3 fractures compared to 41% in the Netherlands [16]. Patients with functionally stable fractures have a shorter rehabilitation time

The aim of this study is to evaluate the clinical outcomes, survival probabilities, and treatment modalities of patients over the age of 50 with AO A1–A4 thoracolumbar fractures. The study focuses on comparing the effectiveness of conservative management, PVP, and surgical stabilization (open surgery and minimally invasive spine surgery) in stable and unstable fracture groups, while also analyzing factors influencing treatment decisions and long-term outcomes.

## 2. Materials and Methods

This retrospective study analyzed the clinical data of 3409 patients treated for thoracolumbar vertebral fractures at Hungary’s largest trauma center between November 2014 and October 2019. The time-to-treatment was defined as the period between the event that triggered the patient’s symptoms and the patient’s hospital admission. The follow-up period began after the fracture was confirmed by computed tomography (CT) and lasted for at least one year for conservatively treated patients, and one year post-surgery for those treated surgically. For deceased patients, the follow-up ended at the time of death, with death data provided by the National Health Insurance Fund of Hungary.

**Figure 1:**
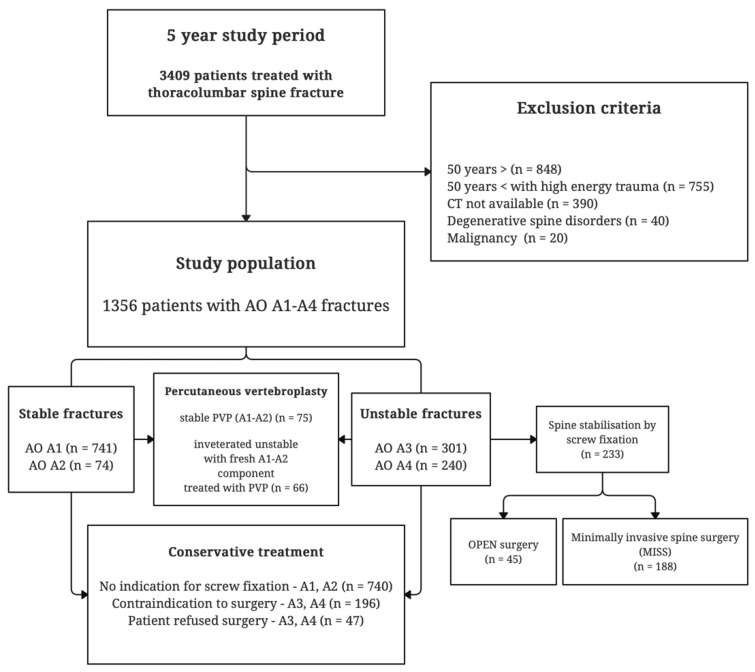
Study design flowchart. Visual representation of the study population and grouping according to fracture type and treatment modality.

Fracture severity was assessed using hospital records and CT scans, and fractures were categorized according to the AO Spine Classification System. As bone mineral density (BMD) values were not available for all patients, osteoporotic status was inferred based on the patient’s age and the circumstances of injury. Inclusion criteria were patients aged over 50 with AO A1–A4 type spinal fractures caused by low-impact trauma. Exclusion criteria included the absence of a CT scan, the presence of primary or secondary spinal malignancy, and degenerative spinal conditions such as ankylosing spondylitis and diffuse idiopathic skeletal hyperostosis (Figure 1). These conditions were excluded because CT scans alone are insufficient for distinguishing between AO A3–A4 and AO Type-B fractures, which require additional MRI scans for proper classification.

Of the initial 3409 patients, 2053 were excluded due to factors such as being under 50 years old, sustaining high-energy trauma, having degenerative spine conditions or spinal malignancies, or lacking the high-resolution CT scans necessary for accurate AO spine fracture classification. After applying the inclusion criteria, a total of 1356 patients with AO A1–A4 fractures were included in the final study.

Survival Analysis and Patient Groups:

The primary outcome of this study was to investigate the factors influencing the survival rate of patients with thoracolumbar fractures. Survival probabilities were estimated using Cox proportional hazards models to evaluate the effects of different treatment modalities and patient characteristics

The following key factors were analyzed to determine their impact on patient survival:

‐ Fracture stability: Patients were categorized into stable (AO A1 and A2) and unstable (AO, A3, and A4) fracture groups.
‐ Treatment modality: Patients received either conservative treatment, PVP, or surgical stabilization using transpedicular screw fixation (either open surgery or minimally invasive spine surgery, MISS).
‐ Other factors: Age, sex, and contraindications to surgery (e.g., anesthesia risks) were also considered as potential influences on survival.

Patient Groups:

The following patient groups were compared for survival analysis:

‐ Stable fractures (A1–A2): These patients were primarily treated conservatively (740 patients) or with PVP (75 patients).
‐ Unstable fractures (A3–A4): Treatment for this group included surgical screw fixation (233 patients), PVP (66 patients), or conservative management (243 patients), with some patients refusing surgery or deemed unfit for anesthesia.

For each patient group, survival curves were generated to visualize the impact of these factors on long-term survival. Cox proportional hazard models were used to compare survival probabilities between these groups and to assess the influence of specific variables such as treatment modality, age, and sex.

Three different analyses were conducted as follows:

‐ Analysis 1: Compared survival probabilities of patients treated with open surgery versus MISS for unstable fractures.
‐ Analysis 2: Examined the differences in survival between stable and unstable fractures, and the effect of conservative treatment vs. surgical stabilization.
‐ Analysis 3: Investigated the likelihood of receiving surgical stabilization based on the time period (OPEN or MISS period), patient characteristics, and fracture type.

All analyses controlled for confounding factors such as age, sex, and time-to-treatment to ensure the results reflected the true impact of these variables on patient outcomes.

Patient Treatment Protocols:

The most stable AO A1–A2 fractures were treated conservatively. If a patient reported a Visual Analogue Scale (VAS) pain score higher than 3 out of 10 for 4 weeks after starting conservative treatment, PVP was performed to manage persistent pain. Conservative treatment included the use of an external brace, pain management, and physiotherapy.

For unstable AO A3–A4 fractures, screw fixation was the preferred treatment when patients were eligible for surgery and provided consent. This was supplemented with pain management and bracing. However, some patients with unstable fractures refused surgery, even though they were medically fit for general anesthesia and the procedure. The majority of patients with unstable fractures who were treated conservatively were considered ineligible for surgery due to anesthesia-related risks, as assessed by institutional protocols. Patients with a “Revised Cardiac Risk Index for Pre-Operative Risk” exceeding 10% for major adverse cardiac events (MACE) were contraindicated for general anesthesia and were treated conservatively instead.

PVP was performed for inveterate unstable AO A3–A4 fractures that had a fresh AO A1–A2 component causing unremitting pain (these patients formed the “inveterate unstable with fresh A1–A2 component, PVP group.

During the study period, MISS was introduced at our trauma center. For patients requiring screw fixation, open surgery (the OPEN group) was used during the first two years of the study (November 2014 to October 2016). In the last two years (November 2017 to October 2019), only MISS was used (the MISS group). In the third year (November 2016 to October 2017), this period served as a learning phase for MISS at our clinic. Both open and minimally invasive techniques were used. All surgeries were performed by the same surgical team in the same environment, and the patient population remained consistent throughout the study. We compared the accessibility and outcomes of surgical screw fixation between the first and last two years, assuming that the only variable difference was the surgical technique.

## 3. Results

A total of 3409 patients with thoracolumbar fractures were treated during the five-year study period. Of these, 1356 patients (39.8%) met the inclusion criteria. The study group comprised 1071 women (79%) and 285 men (21%), with a mean age of 74.5 years (SD = 10.5) (Table 1). Vertebral fractures were classified as AO A1 (*n* = 741), AO A2 (*n* = 73), AO A3 (*n* = 301), and AO A4 (*n* = 241). The stable fracture group (A1 and A2) included 814 patients, while the unstable group (A3 and A4) had 542 patients. Among the patients, 141 underwent PVP and 233 received transpedicular screw fixation. The median length of hospital stay was 2 days (IQR 3) for stable fractures and 4 days (IQR 5) for unstable fractures, with an overall median stay of 3 days (IQR 2) (Table 1).

### 3.1. Analysis 1: Comparison of OPEN vs. MISS Surgical Stabilization

**Figure 2:**
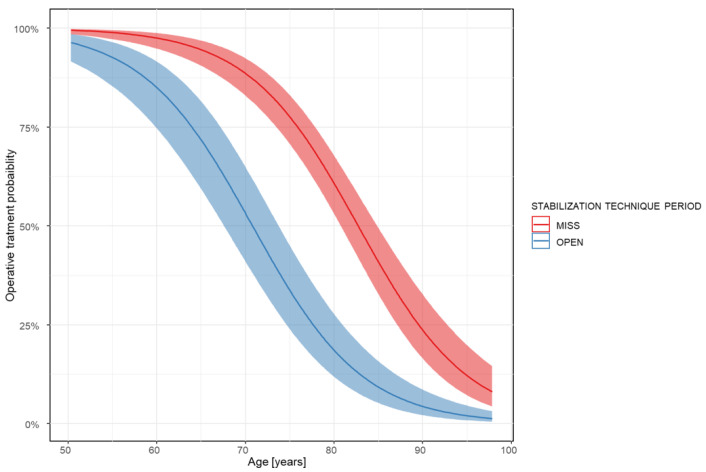
Surgical technique and the probability of surgical care in relation to the patients’ age.

In the first two years (the OPEN period), 124 patients with unstable fractures were treated, and 45 patients (36%) underwent surgical screw fixation. In the last two years (the MISS period), 288 patients with unstable fractures were treated, and 188 patients (65%) underwent surgical screw fixation. The average age of patients receiving surgery was 67.44 years (SD = 9.07) during the OPEN period and 72.24 years (SD = 8.76) during the MISS period. The analysis showed that both age (OR 0.87, CI 0.82 to 0.88; *p* < 0.001) and stabilization technique (OR 0.25, CI 0.08 to 0.26; *p* < 0.001) significantly influenced whether surgery occurred. There was no significant difference in mortality between the MISS and OPEN techniques (OR 0.91, CI 0.44 to 1.89; *p* = 0.79) (Figure 2).

### 3.2. Analysis 2: Survival Outcomes for Stable vs. Unstable Fractures

**Figure 3:**
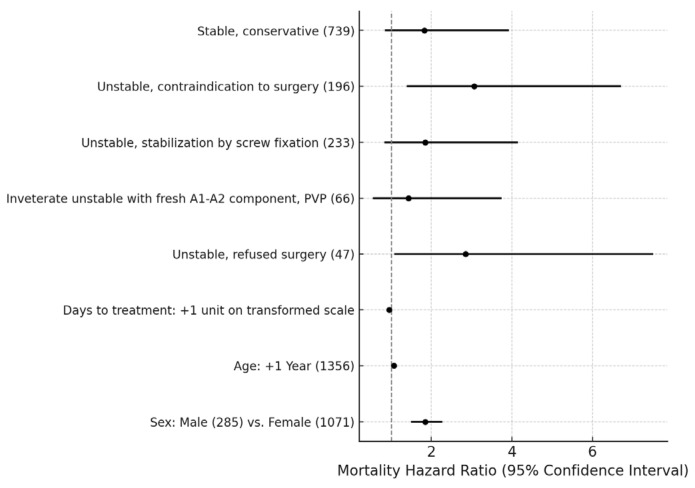
Forest plot showing mortality hazard ratios with 95% confidence intervals for different treatment groups, days to treatment, age, and sex. The vertical dashed line represents a hazard ratio of 1.

**Figure 4:**
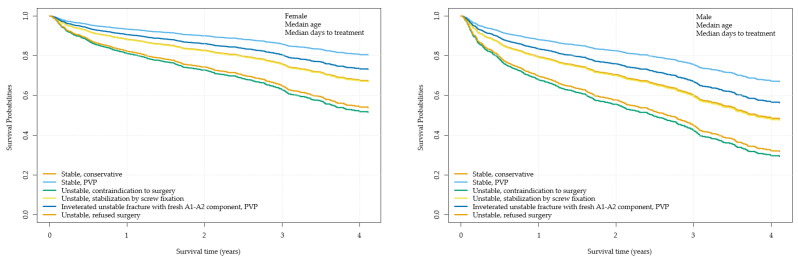
Survival probabilities in relation to time; visual representation of the statistical model. Patients with stable fractures or patients with stabilized fractures have a higher survival probability than patients with unstable fractures treated.

Patients treated conservatively due to contraindications to surgery had a significantly higher mortality hazard ratio (MHR 3.06, CI 1.39 to 6.71; *p* = 0.0013), as did patients who refused surgery (MHR 2.85, CI 1.08 to 7.51; *p* = 0.0272). Unstable fractures treated with screw fixation showed no significant difference in survival compared to the stable PVP reference group (MHR 1.85, CI 0.83 to 4.15; *p* = 0.2242). In contrast, stable fractures treated conservatively showed no significant increase in mortality compared to the reference group (MHR 1.82, CI 0.84 to 3.92; *p* = 0.2060) (Figure 3). Patients with unstable fractures (A3–A4) treated conservatively or with surgery had lower long-term survival rates compared to those with stable fractures (A1–A2) (Figure 4).

### 3.3. Analysis 3: Factors Influencing the Likelihood of Surgery

A logistic regression model was used to assess the likelihood of surgical stabilization during the OPEN and MISS periods. The analysis revealed that older age reduced the likelihood of undergoing surgery (OR 0.87, CI 0.82 to 0.88; *p* < 0.001), and the MISS technique significantly increased the chances of surgical stabilization compared to the OPEN technique (OR 0.25, CI 0.08 to 0.26; *p* < 0.001). Sex did not significantly influence the odds of receiving surgery (OR 0.87, CI 0.39 to 1.44; *p* = 0.58). Furthermore, the median length of hospitalization was shorter for patients treated with MISS (6 days, IQR 4) compared to open surgery (10 days, IQR 7) (Table 2).

### 3.4. General Results on Mortality and Influencing Factors

Regardless of the treatment modality, male patients had a significantly higher mortality hazard ratio than female patients (MHR 1.85, CI 1.50 to 2.27; *p* < 0.001). Older age was also associated with higher mortality. Cox model-based survival curves demonstrated that patients with unstable fractures treated conservatively or with screw fixation had worse long-term survival compared to patients with stable fractures.

## 4. Discussion

In this study, we investigated the impact of fracture severity, as classified by the AO Spine Classification System, and the subsequent treatment strategies on the survival rates of patients with fragility fractures.

This study was an observational analysis aimed at estimating the survival outcomes associated with different treatment modalities for thoracolumbar fractures. It is important to note that the findings of this study should not be interpreted as treatment guidelines or recommendations for managing various types of fractures.

Previous studies, such as Cooper’s, have emphasized that some fractures like osteoporotic fractures are more likely to result from pre-existing comorbidities than to cause death directly [17]. However, our data suggest that while comorbidities undoubtedly increase mortality, the presence of unstable fractures further compounds this risk. For example, patients with unstable fractures who refused surgery had an MHR nearly three times higher than stable PVP-treated patients. This finding highlights the critical importance of spinal stabilization, even for patients who are otherwise suitable for surgery.

While the exact reasons for the higher mortality rate in spinal fracture patients compared to the general population are unclear, it may be attributed to underlying comorbidities and complications related to extended hospitalizations [18,19].

Our use of PVP was guided by clinical indications, such as severe, localized spinal pain without radicular symptoms. Notably, we found no significant difference in the MHR between stable fractures treated conservatively, unstable fractures with fresh AO A1–A2 components treated with PVP, and stable fractures treated with PVP. This suggests that PVP may provide symptomatic relief and stabilization in specific clinical contexts.

Interestingly, we observed that minimally invasive screw fixation achieved comparable mortality outcomes to open surgery, which aligns with other research demonstrating that MISS offers similar clinical and radiological results with reduced trauma, pain, and recovery time [20,21,22]. Although surgical technique (MISS vs. OPEN) did not significantly affect mortality in our study, MISS allowed for the stabilization of a greater number of patients, particularly older ones, compared to the OPEN technique. This is important, as elderly patients with unstable fractures were more likely to undergo MISS, with a greater percentage of patients aged over 70 years treated during the MISS period.

The shorter hospitalization time associated with MISS compared to OPEN surgery (a median of 6 days vs. 10 days) further supports the clinical advantages of MISS in frail, elderly patients. These findings mirror similar observations in hip fracture surgery, where early intervention has been associated with better outcomes, including lower 1-year mortality rates [23,24].

MISS was increasingly favored over open surgery due to its lower invasiveness, reduced recovery time, and suitability for a broader range of patients, particularly the elderly. Despite being less invasive, MISS offers similar or even superior outcomes compared to open surgery, particularly in terms of postoperative recovery and eligibility for surgery in frail patients. These benefits align with modern surgical practices that aim to optimize patient recovery, minimize risks, and improve overall outcomes, especially in vulnerable populations.

The comparison between OPEN and MISS techniques was limited by the smaller sample size in the OPEN group, and we were unable to differentiate between deaths directly related to thoracolumbar fractures and those from other causes. Despite these limitations, the large sample size and inclusion of a broad age range strengthen the reliability of our findings.

From a clinical perspective, comparisons between MISS and mini-open techniques have been evaluated in the literature. One prospective study involving 110 patients demonstrated that the mini-open approach resulted in a long-term improvement of 15.8 degrees in local kyphosis correction and 5.8 degrees in regional Cobb angle correction, compared to 15.4 and 5.5 degrees, respectively, in the MISS group. These findings highlight the nuanced differences in outcomes between the two techniques, which may guide future research and clinical decision making [25].

Our study had some limitations that should be noted. First, as a retrospective observational study, we were unable to assess the independent impact of comorbidities using standardized scoring systems like the Charlson Comorbidity Index or ASA score. Furthermore, we were unable to collect data on the mechanism of injury, which could have provided valuable insights into the natural history of fractures and the management strategies adopted in our cohort. Additionally, we were unable to assess bone mineral density (BMD). However, to exclude non-osteoporotic fractures, we restricted our analysis to patients aged 50 years or older, a criterion commonly used in previous epidemiological studies on osteoporotic spinal fractures [26,27,28].

Despite its limitations, our study provides significant insights into the survival rates with different management strategies of thoracolumbar fractures, particularly in an elderly, frail population. It is one of the few large-scale studies that investigates the impact of fracture stability and surgical technique on patient survival, providing valuable data in an area where the literature is lacking. The inclusion of both MISS and open surgical techniques offers a comprehensive comparison of treatment modalities, reflecting real-world clinical practices.

Our study also spans a substantial five-year period, allowing us to capture long-term outcomes and trends in treatment evolution. Furthermore, the findings highlight the benefits of MISS in reducing recovery time and enabling surgery in older patients, providing evidence to support the broader adoption of less invasive techniques in similar patient populations. Overall, we believe that the study contributes to improving our understanding of the factors influencing survival in patients with spinal fractures and offers practical guidance for optimizing patient care.

## Figures and Tables

**Table 1:** Summary of patient demographics, fracture classifications, treatments, and hospital stays.

Patients Overview	
Patients meeting inclusion criteria	1356 (39.8%)
Women in study group	1071 (79%)
Men in study group	285 (21%)
Mean age of patients	74.5 years (SD = 10.5)
Fracture Classification	
Stable fractures (A1 and A2)	814
Unstable fractures (A3 and A4)	542
Treatments	
Percutaneous Vertebroplasty	141
Screw fixation	233
Hospital Stay	
Median stay for stable fractures	2 days
Median stay for unstable fractures	4 days

SD: Standard Deviation.

**Table 2:** Factors affecting the odds of surgical stabilization and median hospitalization times for MISS and OPEN techniques.

Variable	Odds Ratio	Additional Details
Age	0.87 (CI 0.82 to 0.88)	Older age reduces the chance of surgery
Technique (MISS vs. OPEN)	0.25 (CI 0.08 to 0.26)	MISS increases the chance of surgery
Sex	0.87 (CI 0.39 to 1.44)	Sex did not influence the surgery odds
Median MISS hospitalization	N/A	6 days
Median OPEN hospitalization	N/A	10 days

N/A: Not Applicable.

## Data Availability

The anonymized dataset on which the statistical analysis is based is not publicly available but will be provided upon request to the editorial board.
